# Long noncoding RNA TMPO-AS1 upregulates BCAT1 expression to promote cell proliferation in nasopharyngeal carcinoma via microRNA let-7c-5p

**DOI:** 10.1186/s41021-024-00308-6

**Published:** 2024-06-27

**Authors:** Huan Wang, Fuming Zhou, Jia Wan, Hong Yu, Jin Wang

**Affiliations:** Department of Otolaryngology, Affiliated Hospital of Yunnan University (Second People’s Hospital of Yunnan Province, Yunnan Eye Hospital), 176 Qingnian Road, Kunming City, Yunnan Province 650021 China

**Keywords:** TMPO-AS1, Let-7c-5p, BCAT1, Nasopharyngeal carcinoma, Proliferation, Apoptosis

## Abstract

**Background:**

Long non-coding RNA (lncRNA) is a group of RNA transcripts that contribute to tumor development by post-transcriptionally regulating cancer-related genes. Nasopharyngeal carcinoma (NPC) is an epithelial tumor that occurs in the nasopharynx and is common in North Africa and Southeast Asia. The study investigated the functions of lncRNA TMPO-AS1 in NPC cell proliferation and apoptosis as well as its related competing endogenous RNA (ceRNA) mechanism.

**Methods:**

Candidate microRNA and genes that may regulated by TMPO-AS1 were predicted with the bioinformatic tool starBase. TMPO-AS1 expression in NPC tissue, cells, nuclear part, and cytoplasmic part was measured by RT-qPCR. MTT assay, EdU assay, and flow cytometry analysis were carried out to evaluate NPC cell viability, proliferation, and apoptosis, respectively. RNA immunoprecipitation assay and luciferase reporter assay were conducted to detect the binding between TMPO-AS1 and let-7c-5p or that between let-7c-5p and BCAT1.

**Results:**

TMPO-AS1 and BCAT1 showed high expression in NPC tissue and cells, while let-7c-5p was downregulated in NPC. The silencing of TMPO-AS1 suppressed NPC cell proliferation while promoting cell apoptosis. Moreover, TMPO-AS1 interacted with let-7c-5p and negatively regulated let-7c-5p expression. BCAT1 was a target of let-7c-5p and was inversely regulated by let-7c-5p in NPC cells. The repressive impact of TMPO-AS1 knockdown on NPC cell growth was countervailed by overexpressed BCAT1.

**Conclusion:**

TMPO-AS1 accelerates NPC cell proliferation and represses cell apoptosis by interacting with let-7c-5p to regulate BCAT1 expression.

**Supplementary Information:**

The online version contains supplementary material available at 10.1186/s41021-024-00308-6.

## Introduction

Nasopharyngeal carcinoma (NPC) originates from nasopharynx epithelium and is frequently diagnosed in Southeast Asia and North Africa [1, 2]. According to the data of the International Agency for Research on Cancer, there were approximately 133,354 newly diagnosed NPC cases worldwide in 2020 [3]. Risk factors for NPC include Epstein-Barr virus infection, genetic predisposition, smoking and consumption of salt-cured food [4]. While radiotherapy and chemotherapy have improved patient outcomes, recurrence and distant metastases remain significant challenges for patients with NPC [5]. Targeted therapy holds promise for treating metastatic or recurrent NPC [5], underscoring the importance of identifying differentially expressed genes and exploring mechanisms underlying NPC pathogenesis to enhance diagnostic methods and therapeutical options.

Long noncoding RNAs (lncRNAs) are conserved noncoding RNAs consisting of over 200 nucleotides [6]. Increasing evidence suggests that lncRNAs play an active role in various types of cancer, including NPC. For example, knockdown of lncRNA TUG1 can suppress NPC development by inhibiting epithelial-mesenchymal transition [7]. LncRNA ZFAS1 accelerates NPC progression by activating Wnt/β-catenin pathway [8]. Revealed evidence has shown that overexpression of TMPO-AS1 is associated with exacerbated tumor progression and unfavorable prognosis in patients with prostate cancer [9]. Additionally, TMPO-AS1 promotes cellular phenotypes in cervical cancer by regulating RAB14 via miR-577 [10]. Moreover, TMPO-AS1 facilitates lung carcinogenesis by regulating its antisense transcript TMPO [11]. In NPC, TMPO-AS1 was reported to regulate aggressiveness-related features in NPC by interacting with miR-320a [12]. No more articles were available on the role and mechanism of TMPO-AS1 in NPC. The current study aims to further investigate the potential mechanisms underlying TMPO-AS1 and deepen our understanding of its role in NPC progression.

During cancer progression, lncRNA has been identified as competing endogenous RNA (ceRNA) that interact with microRNA (miRNA), thereby sequestering messenger RNA (mRNA) from miRNA-mediated repression [13, 14]. MiRNAs are transcripts with 20–24 nucleotides in length and belong to small noncoding RNAs. MiRNA can bind to 3′-untranslated region (UTR) of mRNA and thereby suppress mRNA translation [15]. The ceRNA hypothesis centered on TMPO-AS1 has been documented in ovarian cancer [16], osteosarcoma [17], retinoblastoma [18], lung cancer [19, 20], and gastric cancer [21]. Previous research confirmed the significant influence of miRNAs on the pathological progression of malignancies [22]. For example, miR-324-3p restrains cell migration by inversely regulating WNT2B in NPC [23]. Overexpressed miR106A-5p inhibits autophagy and promotes malignant phenotypes including cell proliferation and metastasis in NPC [24]. In the current study, the miRNAs and genes involved in the ceRNA network mediated by TMPO-AS1 were investigated. MicroRNA let-7c-5p was identified to be the downstream miRNA of TMPO-AS1. Although the tumor-suppressing role of let-7c-5p is identified in many tumors, its functions and mechanism in NPC is unknown. In addition, BCAT1, in the current study, is demonstrated as the target of let-7c-5p. BCAT1 was previously reported to act as an oncogene in NPC [25].

In conclusion, the present study explored the role of TMPO-AS1 in NPC cell growth and related the ceRNA network. The study verified the oncogenic role of TMPO-AS1 in driving NPC progression via the let-7c-5p/BCAT1 axis, indicating that TMPO-AS1 might be a promising candidate for targeted therapy in NPC.

## Materials and methods

### Sample collection

NPC tissue (*n* = 12) and adjacent normal tissue (*n* = 12) were collected from NPC patients at Affiliated Hospital of Yunnan University. All the samples were quickly kept at -80℃. None of the patients had received any anticancer treatment before surgery. All participants signed informed consents, and the study was under approval of Ethics Committee of Affiliated Hospital of Yunnan University.

### Cell lines and cell culture

NPC cell lines (5-8F, CNE-1, CNE-2, and SUNE-1) and normal human nasopharyngeal epithelial cell line (NP69) were obtained from the Chinese Academy of Science Cell Bank (Shanghai, China). The above cells were cultured in DMEM (Gibco, USA) containing 10% fetal bovine serum in an incubator containing 5% CO_2_ and set at 37℃.

### Transfection

The plasmids used in the study were small interfering RNA against TMPO-AS1 (si-TMPO-AS1), let-7c-5p mimics, and pcDNA3.1 vectors containing full sequence of BCAT1 (pcDNA3.1/BCAT1), which were used to silence TMPO-AS1, overexpress let-7c-5p, and upregulate BCAT1 expression, respectively. These plasmids and their corresponding negative controls (si-NC, NC mimics, pcDNA3.1 vector) were obtained from GenePharma (Shanghai, China). For cell transfection, the plasmids were then transfected into 5-8F and CNE-1 cells using Lipofectamine 2000 (Invitrogen, Carlsbad, USA) for 48 h.

### Subcellular RNA fractionation assay

The nuclei Isolation Kit (Sigma Aldrich, St. Louis, USA) was applied for the subcellular fractionation assay according to the manufacturer’s recommendations. The extracted nuclear or cytoplasmic RNA was subjected to RT-qPCR analysis for measurement of TMPO-AS1, GAPDH, and U6 in each part.

### Reverse transcription quantitative PCR

First, RNA extraction from cultured cells was performed using TRIzol reagent (Invitrogen) following the manufacturer’s instruction. Next, Reverse Transcription Kit (Toyobo, Osaka, Japan) was applied to convert total RNA to cDNA. PCR was conducted utilizing TB Green Premix Ex Taq (Takara, Japan) on ABI PCR system (Applied Biosystems, Foster City, USA). GAPDH was the internal control for TMPO-AS1 and mRNAs, and U6 was the reference for miRNAs. RNA expression levels were calculated using the 2^−ΔΔCt^ method.

### Western blotting

After cells were lysed by RIPA buffer, the protein was extracted using 12% SDS-PAGE and then transferred onto the PVDF membrane. Next, the membrane was blocked with 5% fat-free milk and then incubated with primary antibodies against BCAT1 (ab232700, 1:500) and GAPDH (ab8245) overnight at 4℃. Then the membrane was cultured for 2 h at 37℃ with secondary antibodies. An ECL kit (GE Healthcare, Chicago, USA) was applied to visualize the bands, and the intensity was analyzed using ImageJ software (National Institutes of Health, Bethesda, USA).

### Methyl thiazolyl tetrazolium (MTT) assay

MTT assays were carried out to examine the viability of cancer cells after indicated treatment. After plated onto 96-well plates, NPC cells (5 × 10^3^ per well) was cultured for 24 h, 48 h, or 72 h. At each timepoint, cells were supplemented with the medium containing 100 μg MTT (Sigma Aldrich) for 4 h of incubation at 37℃. Then cells were treated with 100 μl DMSO for 10 min, and a microplate reader (Bio-Rad, Hercules, USA) was applied to determine the absorbance at 490 nm wavelength.

### Detection of cell proliferation

EdU assays were utilized to assess cell proliferation with the EdU detection kit (Ribobio, Guangzhou, China). After transfection, cells were treated with EdU solution (50 μmol/L) for 2 h at 37℃. Next, 4% paraformaldehyde was added to fix cells for 30 min. Then, anti-EdU working solution was supplemented. The nuclei of cells were labelled with DAPI. A fluorescence microscopy (Leica, Germany) was used to observe and calculate the percentage of EdU-positive cells.

### TUNEL assay

Cell apoptosis after indicated treatment was evaluated by TUNEL (Terminal deoxynucleotidyl transferase-mediated dUTP nick-end labeling) assay using an in-situ apoptotic cell detection kit (Takara, Beijing, China). A microscope (Leica, Germany) was used to capture the images at five randomly selected sections.

### Flow cytometric analysis

Flow cytometric analysis was applied to analyze cell apoptosis using Annexin V Kit (Beyotime, China). Cells were reaped after 48 h and washed in PBS twice. Then, propidium iodide (PI) and Annexin V-FITC were utilized to double stain the NPC cells (1 × 10^6^ cells/mL). The CellQuest software (Becton, Dickinson and Company, USA) was utilized for data analysis.

### Luciferase reporter assay

The pmirGLO vectors containing TMPO-AS1-wild type (Wt), TMPO-AS1-mutated (Mut), BCAT1-Wt or BCAT1-Mut sequence were synthesized by RioBio (Guangzhou, China). These vectors were co-transfected together with let-7c-5p mimics or NC mimics into CNE-1 and 5-8F cells using Lipofectamine 2000 (Invitrogen). Forty-eight hours later, the firefly luciferase activity and Renilla activity were examined with the help of the luciferase reporter assay system (Promega, Madison, USA).

### RNA immunoprecipitation (RIP) assay

RIP kit (Millipore, USA) was used to detect RNA interaction. The lysis buffer was added to cells on ice. Magnetic beads were incubated with 5 μg Ago2 antibody (Abcam, UK) for 30 min. The IgG antibody (Abcam) was set as a control. Afterwards, the cell lysates were added to the RIP buffer containing the beads. RNA on the beads were extracted by proteinase K and subjected to qPCR analysis.

### Statistical analysis

All data are shown as the mean ± standard deviation, and significance was analyzed using Student’s *t*-test (for comparison between two groups) or analysis of variance (for comparison among multiple groups) followed by Tukey’s post hoc analysis. Spearman’s coefficient analysis was utilized to analyze gene expression correlation in NPC tissue samples. Values of *p* < 0.05 were regarded to be statistically significant.

## Results

### TMPO-AS1 is upregulated in NPC tissue and cells

**Fig. 1:**
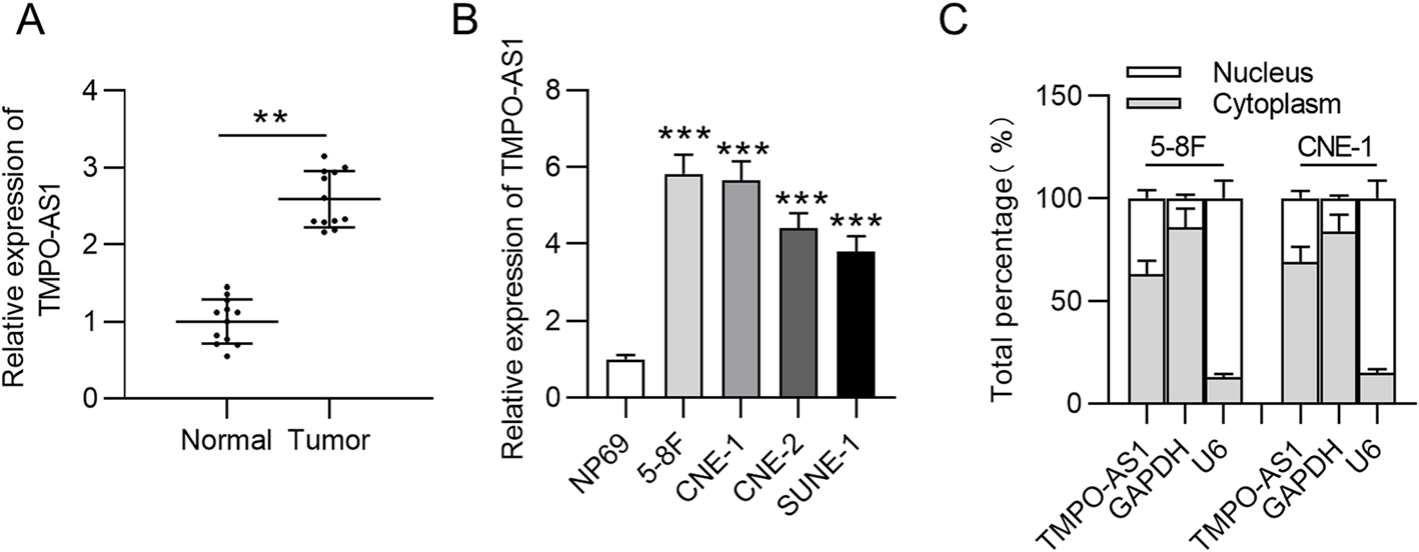
High expression of TMPO-AS1 in NPC tissue and cells. **A** Expression of TMPO-AS1 in NPC tissue (*n* = 12) and adjacent healthy tissue (*n* = 12) was evaluated using PCR. **B** The expression of TMPO-AS1 in human NPC cells and normal human nasopharyngeal epithelial cells (NP69) was determined by PCR. **C** The distribution of TMPO-AS1 in cytoplasmic or nuclear parts of CNE-1 and 5-8F cells was evaluated using subcellular fractionation assay. GAPDH was used as the cytoplasmic control, and U6 was the nuclear control. ^**^p < 0.01, ^***^p < 0.001

RT-qPCR was performed to determine TMPO-AS1 expression in human NPC samples and cell lines. As shown by Fig. 1A, TMPO-AS1 levels were significantly upregulated in tumor samples compared with its expression in corresponding normal samples (**p < 0.01). Consistently, TMPO-AS1 expression was also increased in NPC cell lines, especially in 5-8F and CNE-1 cells (5.82 and 5.65 folds), compared to its expression in normal nasopharyngeal cell line NP69 (Fig. 1B). The findings suggest that TMPO-AS1 might be an oncogene in NPC. According to subcellular RNA fractionation experiments, TMPO-AS1 showed predominant cytoplasmic distribution in 5-8F and CNE-1 cells, as indicated by a higher percentage of TMPO-AS1 expression in the cytoplasm (68% and 72%) than nucleus (Fig. 1C). The results indicated that TMPO-AS1 can regulate genes at the post-transcriptional level and has the potential to be a ceRNA in NPC cells.

### TMPO-AS1 knockdown hampers NPC cell proliferation and induces cell apoptosis

**Fig. 2:**
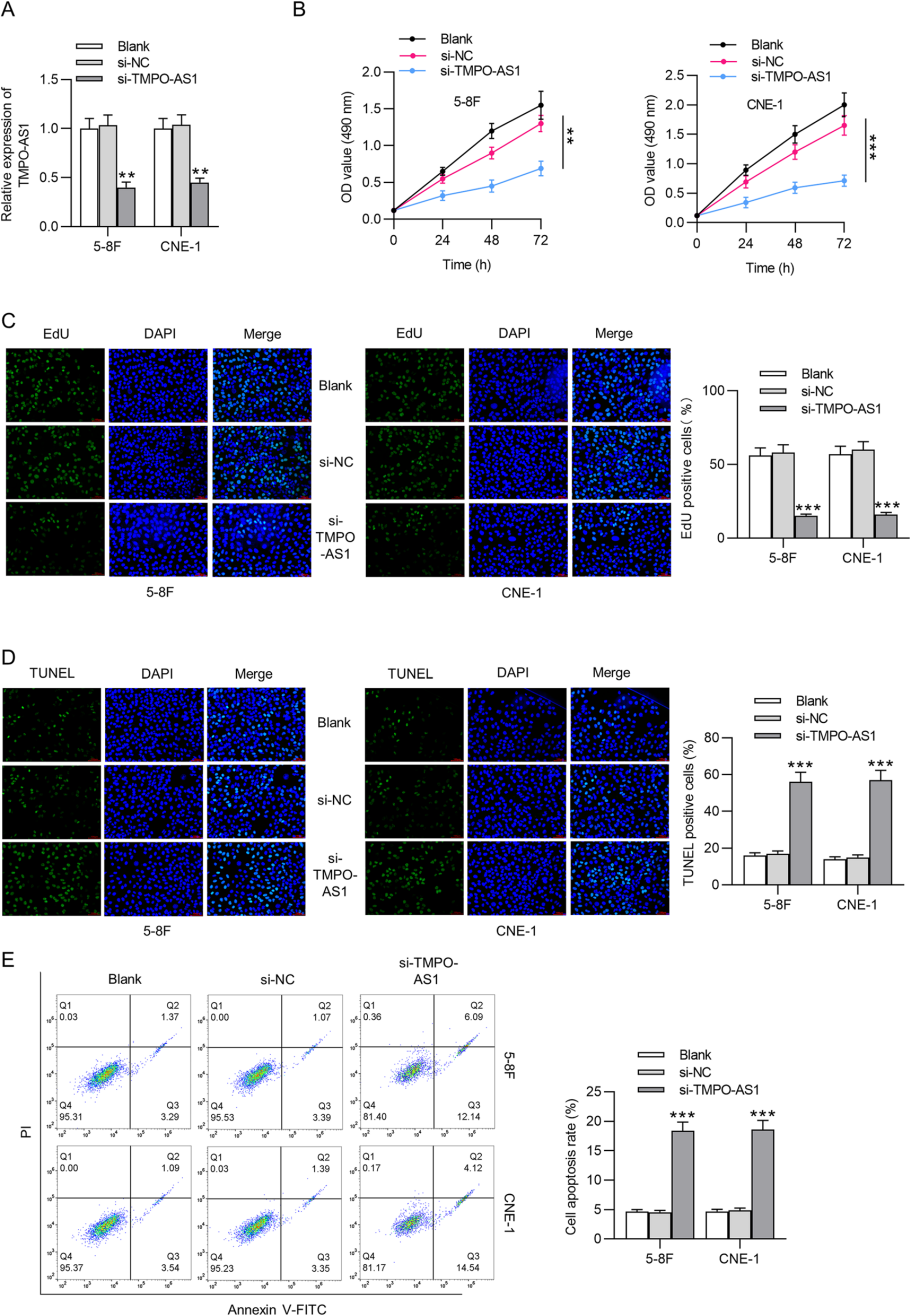
TMPO-AS1 silencing affects NPC cell proliferation and apoptosis. **A** PCR analysis of TMPO-AS1 expression in NPC cells transfected with si-TMPO-AS1 or si-NC. **B**-**C** Cell viability and proliferation were detected through MTT and EdU assays, respectively. **D**-**E** Cell apoptosis was detected through TUNEL assays and flow cytometry analyses. ^**^p < 0.01, ^***^p < 0.001

Functional experiments were performed to identify the biological role of TMPO-AS1 in NPC. Figure 2A revealed that TMPO-AS1 level was successfully reduced in NPC cells (61% and 57% decrease) after transfection of si-TMPO-AS1. Results of MTT assays implied that cell viability was effectively reduced in si-TMPO-AS1 group compared to that in the control group (Fig. 2B, **p < 0.01, ***p < 0.001). Additionally, a prominent decrease in the number of EdU-positive cells was seen after TMPO-AS1 deficiency (15% and 16%) compared with that in the si-NC group (58% and 60%) (Fig. 2C). On the contrary, the proportion of TUNEL-stained cells was noticeably increased in response to TMPO-AS1 knockdown (56% and 57%) compared to apoptotic rate in the control group (17% and 15%). Results of flow cytometry analysis showed that NPC cell apoptotic rate in the si-TMPO-AS1 group was increased to 18.33% and 18.58%, which is higher than that in si-NC group (4.52% and 4.85%) (Fig. 2E).

### TMPO-AS1 interacts with let-7c-5p

**Fig. 3:**
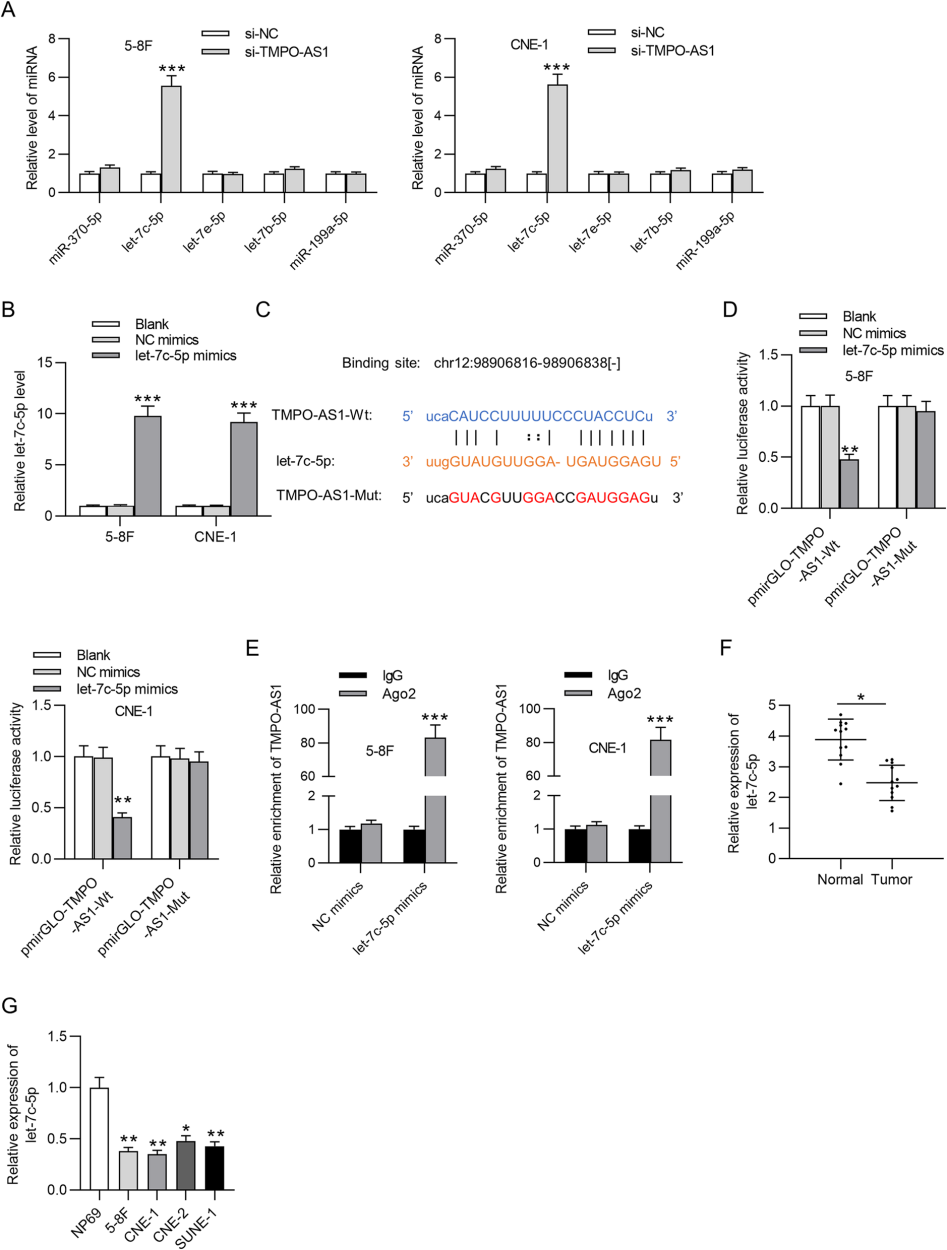
TMPO-AS1 interacts with let-7c-5p in NPC cells. **A** The candidate miRNAs were predicted with the screening conditions: CLIP Data: strict stringency (> = 5), Pan-Cancer: 8 cancer types. The impact of TMPO-AS1 depletion on expression levels of five candidate miRNAs was analyzed using qPCR analysis. **B** PCR analysis of let-7c-5p level in NPC cells transfected with let-7c-5p mimics or NC mimics. **C** Binding site between TMPO-AS1 and let-7c-5p was obtained from starBase. **D** and **E** The binding ability of TMPO-AS1 and let-7c-5p was validated using luciferase reporter assay and RNA immunoprecipitation assay. **F** PCR analysis of let-7c-5p level in NPC tissue and adjacent normal tissue. **G** PCR analysis of let-7c-5p level in NPC cells and normal nasopharyngeal cells (NP69). ^*^p < 0.05, ^**^p < 0.01, ^***^p < 0.001

The bioinformatics tool, starBase v3.0 software, was used to seek for potential miRNA targets of TMPO-AS1 with the criterion of CLIP Data: strict stringency (> = 5), Pan-Cancer: 8 cancer types. Five miRNAs were identified, which are let-7c-5p, let-7e-5p, let-7b-5p, miR-370-5p, and miR-199a-5p. Results of RT-qPCR revealed that only let-7c-5p level was significantly increased in response to TMPO-AS1 depletion (5.56 and 5.63 folds), while expression levels of the rest four genes were not significantly altered (Fig. 3A). Therefore, let-7c-5p was identified for subsequent experiments. Let-7c-5p was overexpressed in NPC cells through transfection of let-7c-5p mimics (9.6 and 9.3 fold increase) (Fig. 3B). The binding area between TMPO-AS1 and let-7c-5p was predicted with starBase, and the mutant sequence of TMPO-AS1 is included in Fig. 3C. Let-7c-5p mimics significantly attenuated pmirGLO-TMPO-AS1-Wt luciferase activity in 5-8F and CNE-1 cells (**p < 0.01), while TMPO-AS1-Mut activity was not significantly altered by let-7c-5p overexpression (Fig. 3D). In addition, overexpressed let-7c-5p led to abundant enrichment of TMPO-AS1 in the Ago2 group compared with that in the IgG group (83.2 and 81.7 folds) (Fig. 3E). The results implied the binding between TMPO-AS1 and let-7c-5p. Moreover, downregulated let-7c-5p level was detected in NPC tissue and cells using PCR analysis (Fig. 3F and 3G, *p < 0.05, **p < 0.01).

### BCAT1 is targeted by let-7c-5p in NPC cells

**Fig. 4:**
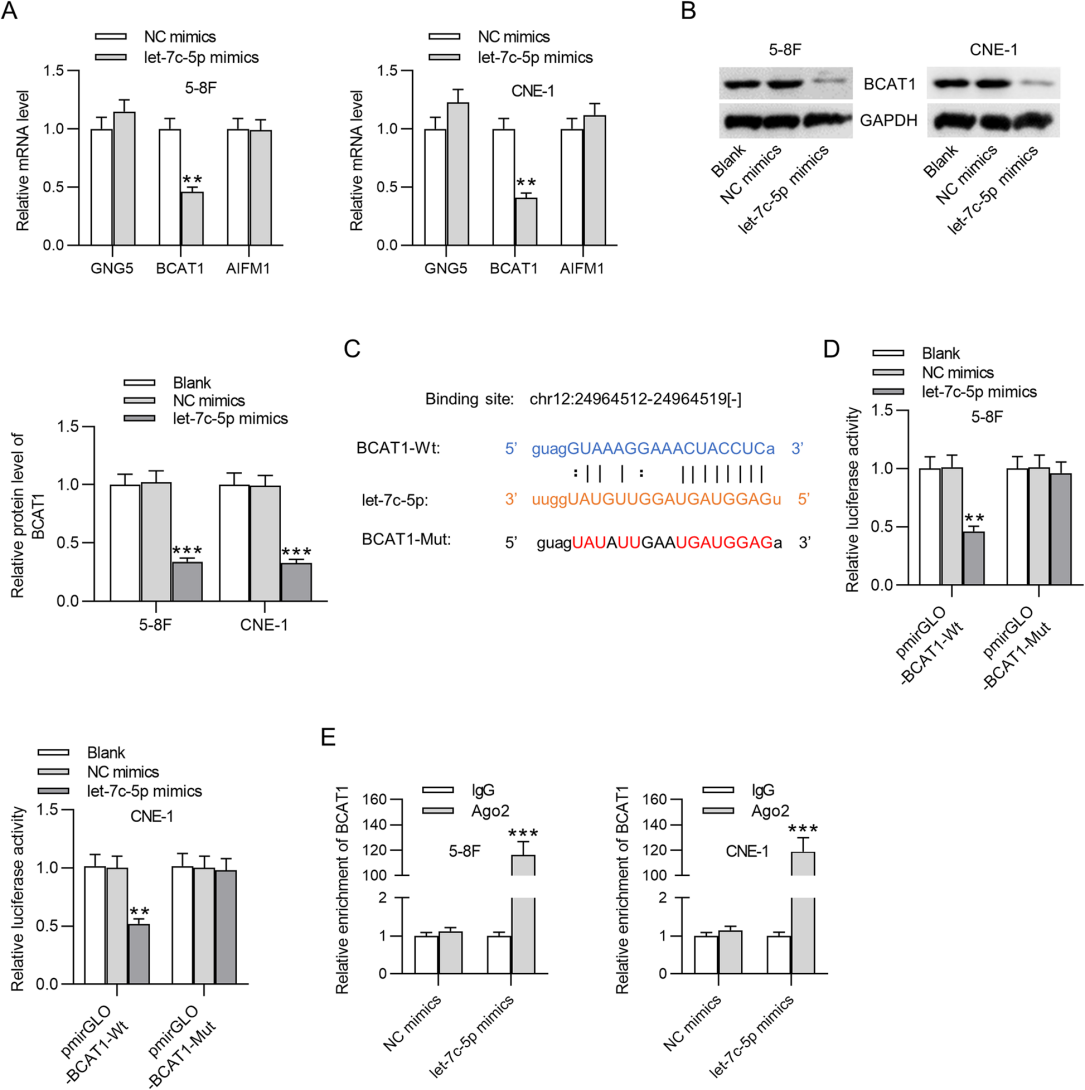
The relationship between let-7c-5p and BCAT1 in NPC. **A** Targets of let-7c-5p were identified with the prediction criterion of CLIP Data: strict stringency (> = 5), Pan-Cancer: 10 cancer types, Degradome: high stringency (> = 3), and Predicted Program: microT + miRanda. The mRNA levels of three genes in NPC cells overexpressing let-7c-5p were evaluated using PCR analysis. **B** Western blotting was conducted for quantification of BCAT1 protein level in NPC cells overexpressing let-7c-5p. **C** Binding area between let-7c-5p and BCAT1 was predicted with the starBase. **D** and **E** Binding capacity between BCAT1 and let-7c-5p was proved by luciferase reporter assays and immunoprecipitation assays. ^**^p < 0.01, ^***^p < 0.001

Target genes of let-7c-5p were identified through the bioinformatic tool starBase v3.0 database under the conditions of CLIP Data: strict stringency (> = 5), Pan-Cancer: 10 cancer types, Degradome: high stringency (> = 3), and Predicted Program: microT + miRanda. Three candidate mRNAs (GNG5, BCAT1, and AIFM1) were identified, and Fig. 4A revealed that only BCAT1 mRNA was effectively lowered by let-7c-5p overexpression in 5-8F cells and CNE-1 cells (54% and 59% decrease). In addition, BCAT1 protein level was also suppressed by let-7c-5p overexpression (Fig. 4B). The binding sequence of BCAT1 and let-7c-5p is shown in Fig. 4C. Figure 4D revealed that let-7c-5p mimics prominently inhibited BCAT1-Wt luciferase activity (54% and 48% decrease) instead of BCAT1-Mut activity (Fig. 4D). Moreover, the abundant enrichment of BCAT1 in let-7c-5p mimics + Ago2 group (116.3 and 119 folds) also confirmed the binding possibility between let-7c-5p and BCAT1 (Fig. 4E).

### BCAT1 displays high levels in NPC tissue and cells

**Fig. 5:**
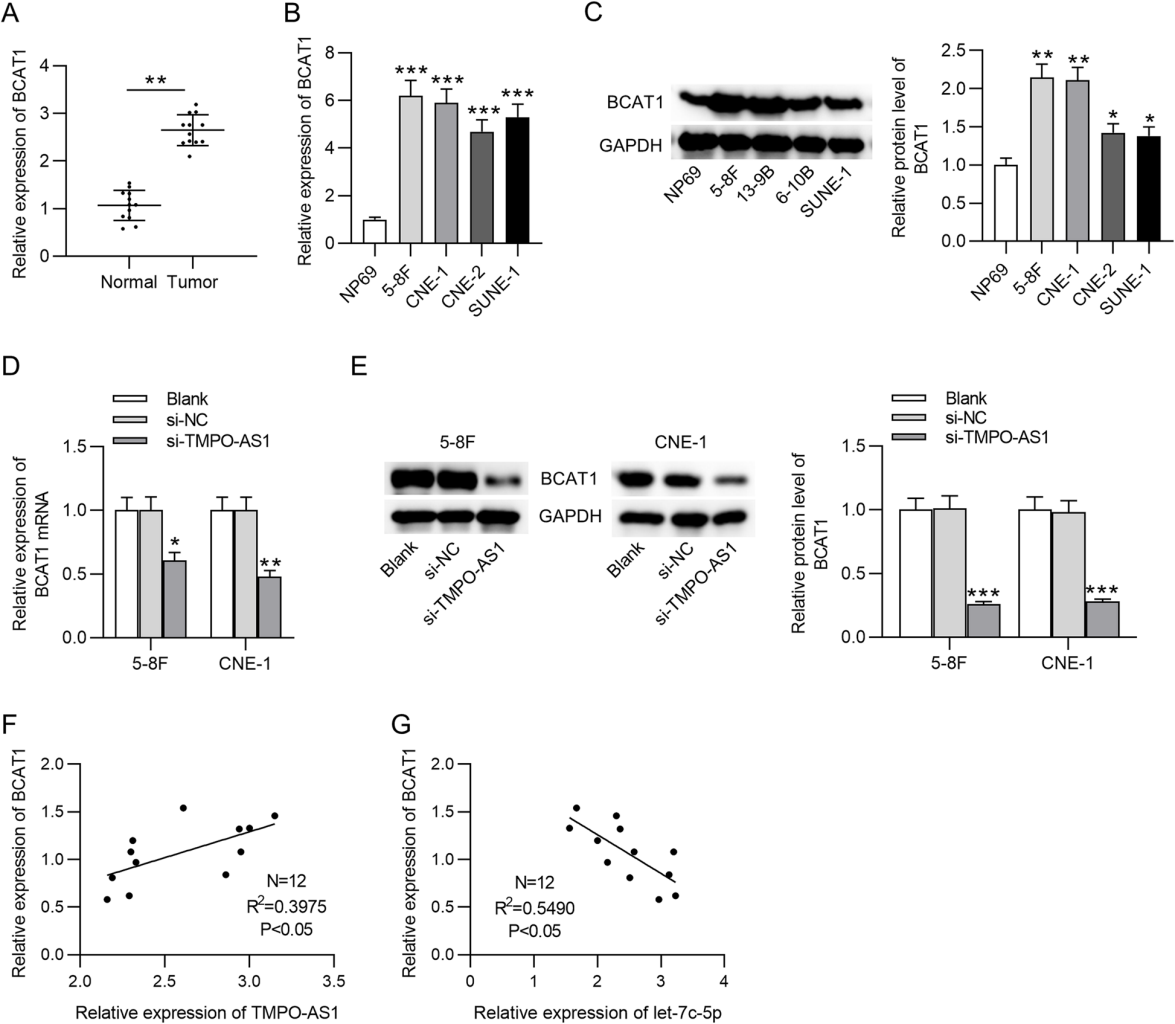
Expression correlation between TMPO-AS1 (or let-7c-5p) and BCAT1. **A** PCR analysis of BCAT1 level in NPC tissue and adjacent normal tissue. **B**-**C** PCR and western blot analyses were performed to measure BCAT1 expression in NPC cells and normal human nasopharyngeal cell line (NP69). **D**-**E** PCR and western blot analyses of BCAT1 mRNA and protein levels in NPC cells silencing TMPO-AS1. The expression correlation (**F**) between TMPO-AS1 and BCAT1 and that (**G**) between let-7c-5p and BCAT1 in NPC tissue was analyzed using Spearman’s correlation coefficient. ^*^p < 0.05, ^**^p < 0.01, ^***^p < 0.001

BCAT1 mRNA expression was significantly elevated in NPC tissue and cells (Fig. 5A and B, **p < 0.01, ***p < 0.001). High BCAT1 expression at the protein level was also detected in NPC cell lines (Fig. 5C). Moreover, the silencing of TMPO-AS1 greatly reduced BCAT1 mRNA and protein levels in NPC cells (Fig. 5D and E). BCAT1 expression was positively correlated to TMPO-AS1 expression, and BCAT1 level was inversely related to let-7c-5p level in NPC tissue samples (Fig. 5F and G).

### TMPO-AS1 promotes cell proliferation and obstructs apoptosis by upregulating BCAT1

**Fig. 6:**
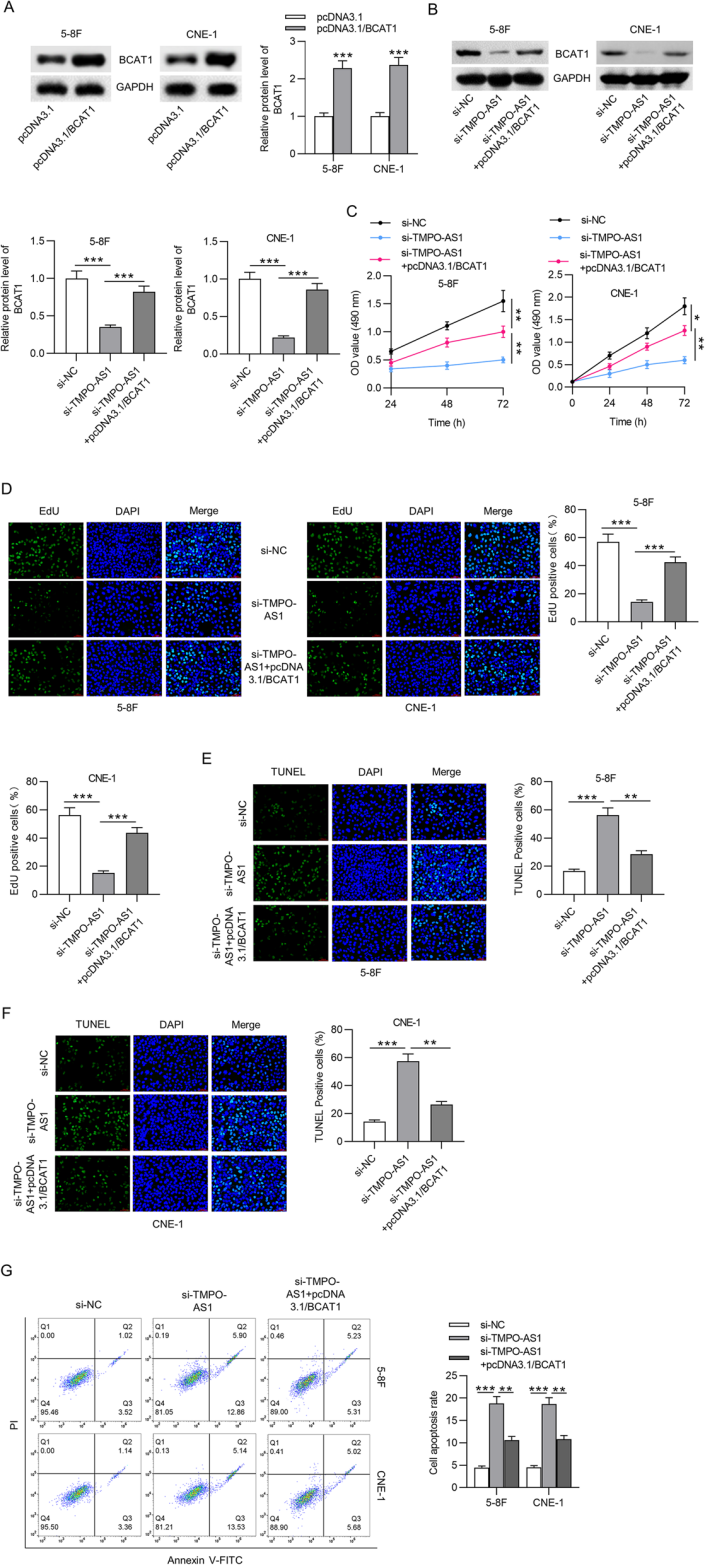
TMPO-AS1 promotes NPC cell proliferation while prohibiting cell apoptosis via BCAT1. **A** The overexpression efficacy of BCAT1 in 5-8F and CNE-1 cells was evaluated by western blot. **B** Western blot analysis of BCAT1 protein level in NPC cells with si-NC, si-TMPO-AS1, or pcDNA3.1/BCAT1 + si-TMPO-AS1. **C**-**D** MTT and EdU assays for cell viability and proliferation detection. **E**–**G** TUNEL assays and flow cytometry analyses for cell apoptosis evaluation. ^**^p < 0.01, ^***^p < 0.001

Rescue experiments were carried out to validate whether TMPO-AS1 promotes NPC cell growth by regulating BCAT1. BCAT1 protein expression was successfully increased in 5-8F and CNE-1 cells after the transfection of pcDNA3.1/BCAR1 (2.28 and 2.37 fold increase) (Fig. 6A). Additionally, the decrease in BCAT1 protein expression induced by si-TMPO-AS1 was rescued by co-transfection with pcDNA3.1/BCAT1 (Fig. 6B). Results of MTT assays reflected that the reduction of cell viability in the context of TMPO-AS1 was rescued by BCAT1 overexpression (Fig. 6C). The reduced number of EdU-stained cells induced by TMPO-AS1 depletion (14.35%, 15.28%) was rescued by BCAT1 upregulation (42.55%, 43.62%) (Fig. 6D). In addition, according to results of TUNEL assays and flow cytometric analyses, TMPO-AS1 depletion led to increased number of TUNEL-positive cells and high cell apoptotic rate, and the alterations were counteracted by BCAT1 upregulation (Fig. 6E-G). These results demonstrated that TMPO-AS1 promoted NPC cell proliferation and repressed apoptosis via upregulation of BCAT1.

## Discussion

Although radiotherapy is currently the preferred treatment for early-stage NPC, the associated toxic effects pose challenges for patients [26]. Targeted therapy is promising for improving outcomes in advanced NPC patients [27]. In recent years, numerous lncRNAs have been discovered to significantly influence the development of various cancers [28, 29]. Notably, lncRNA TMPO-AS1 has been linked to tumor progression and poor prognosis in patients [9–11]. However, its role in NPC remains largely unexplored. The present study revealed upregulation of TMPO-AS1 in NPC tissue and cells. In addition, silencing TMPO-AS1 repressed cell proliferation while enhancing cell apoptosis in NPC. These findings suggest a tumorigenic role of TMPO-AS1 in NPC development. Our data align with previous reports demonstrating the upregulation of TMPO-AS1 expression in thyroid cancer [30], osteosarcoma [31], and bladder cancer [32] and highlighting the anticancer potential of TMPO-AS1 deficiency. Despite our findings on the promoting effect of TMPO-AS1 on cell growth, it has also been reported to accelerate cell migration and invasion in bladder cancer [32] and facilitate bone metastasis in prostate cancer [33]. Although this study did not explore the effect of TMPO-AS1 on other malignant behavior of NPC cells, these aspects can be investigated as future research directions.

Accumulating evidence indicates that lncRNAs can interact with miRNAs to alter gene expression, thus participating in cancer development. For instance, lncRNA AFAP1-AS1 fosters NPC metastasis by acting as a ceRNA of miR-423-5p to control the activation of Rho/Rac signaling [34]. LncRNA HOXC-AS1 accelerates NPC development by interacting with miR-4651 and then upregulating FOXO6 [35]. The bioinformatics tool starBase, also known as the encyclopedia of RNA interactomes (ENCORI), is widely used to predict miRNAs having binding area with a certain lncRNA [36]. Previously, the binding area between lncRNA FGD5-AS1 and miR-195-5p was predicted with the help of starBase [37]. Candidate miRNAs regulated by lncRNA XIST were also predicted with the starBase database [38]. According to our screening criteria, five miRNAs (miR-370-5p, let-7c-5p, let-7e-5p, let-7b-5p, and miR-199a-5p) were identified to be downstream miRNA targets of TMPO-AS1. Experimental results further verified that let-7c-5p was abnormally expressed in response to TMPO-AS1 downregulation. TMPO-AS1 specifically binds to let-7c-5p and negatively regulates let-7c-5p level in NPC cells. The inverse relationship between TMPO-AS1 and let-7c-5p has previously been mentioned in lung cancer, where TMPO-AS1 upregulates STRIP2 expression through its interaction with let-7c-5p [20]. Compared with the previous study, the present work provides a novel target gene of let-7c-5p, which is BCAT1. MiR-let-7c-5p is widely accepted to play the tumor-suppressive role in a variety of diseases. It has been reported that let-7c-5p inhibits colorectal cancer cell proliferation by targeting DUSP7 and inactivating MAPK/ERK signaling [39]. Let-7c-5p represses cell proliferation while accelerating cell apoptosis in breast cancer through inversely regulating ERCC6 [40]. In line with these studies, the current work revealed the downregulation of let-7c-5p in NPC tissue and cells, and it directly targeted the 3’UTR of BCAT1.

MiRNA downregulates the expression of target genes by promoting their degradation or inhibiting their protein translation [41]. Let-7c-5p, in this study, was found to inversely regulate BCAT1 mRNA and protein levels in NPC. BCAT1 is an enzyme responsible for catalyzing the catabolism of branched-chain amino acids [42]. It has been validated to be an oncogene in many cancers. For instance, BCAT1 promotes cell proliferation in glioma through amino acid catabolism [43]. BCAT1 facilitates endometrial cancer cell proliferation through reprogrammed metabolism of branched-chain amino acids [44]. Importantly, c-Myc was revealed to induce high BCAT1 expression and aggravate malignant cellular behaviors in NPC [25]. That may partly explain the high BCAT1 expression in NPC cells. Overall, the existing literature reveals that BCAT1 is a risk factor in many types of cancer. In this study, BCAT1 was discovered to show high expression levels in NPC tissue and cells. Additionally, BCAT1 was demonstrated to be indirectly upregulated by TMPO-AS1. Moreover, overexpressed BCAT1 could countervail the suppressive impact of TMPO-AS1 deficiency on NPC cell growth, indicating that TMPO-AS1 promotes NPC cell proliferation by upregulating BCAT1.

In summary, TMPO-AS1 promotes NPC cell proliferation while restraining cell apoptosis through its interaction with let-7c-5p to regulate BCAT1 mRNA and protein levels, indicating that TMPO-AS1 may act as a promising biomarker for targeted therapies in NPC.

### Supplementary Information

Additional file 1. Supplementary figure 1-3: RNA gel images for subcellular RNA fractionation assay.

## Data Availability

The raw sequence data reported in this paper and detailed information will be available from the authors on request.
