# Supplementary figures and images for: Long noncoding RNA TMPO-AS1 upregulates BCAT1 expression to promote cell proliferation in nasopharyngeal carcinoma via microRNA let-7c-5p
Source: Genes Environ. 2024 Jun 27;46:14. doi: 10.1186/s41021-024-00308-6 (PMC11210057; doi:10.1186/s41021-024-00308-6)

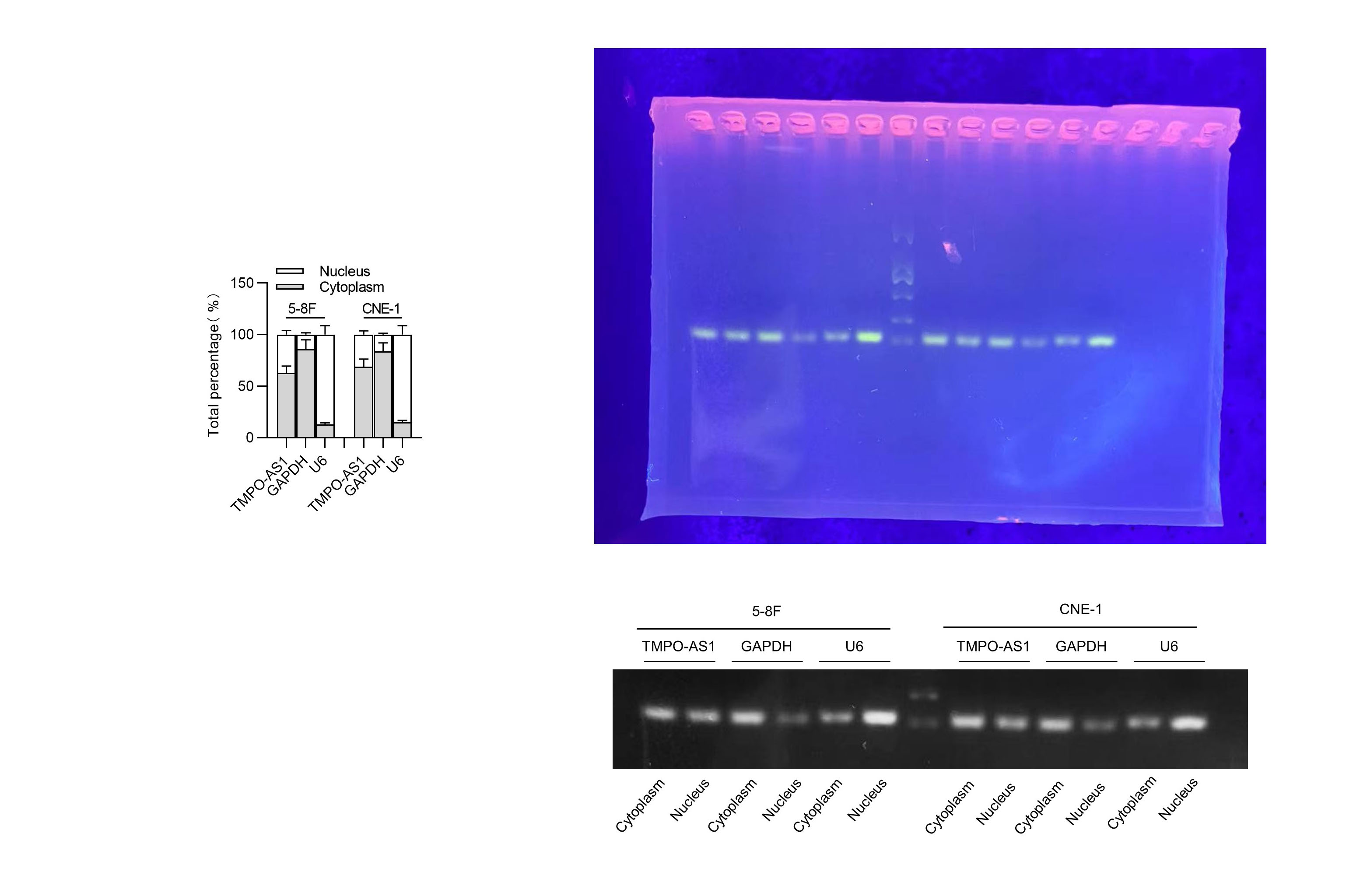

Supplement: Supplementary file 1 — Additional file 1. Supplementary figure 1-3: RNA gel images for subcellular RNA fractionation assay. [file 41021_2024_308_MOESM1_ESM.zip › S Figures/S Figure 1.JPG]

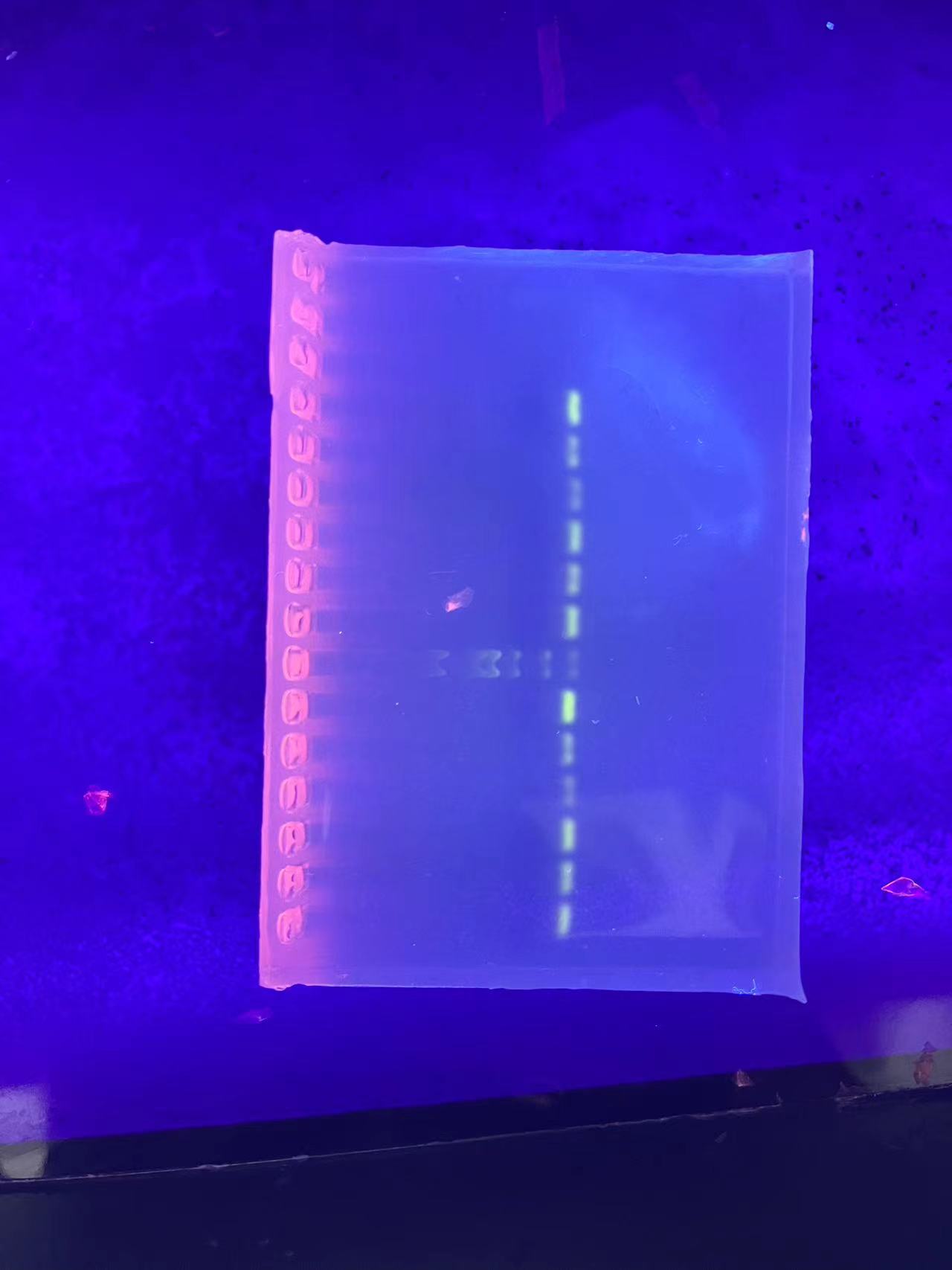

Supplement: Supplementary file 1 — Additional file 1. Supplementary figure 1-3: RNA gel images for subcellular RNA fractionation assay. [file 41021_2024_308_MOESM1_ESM.zip › S Figures/S Figure 2.jpg]

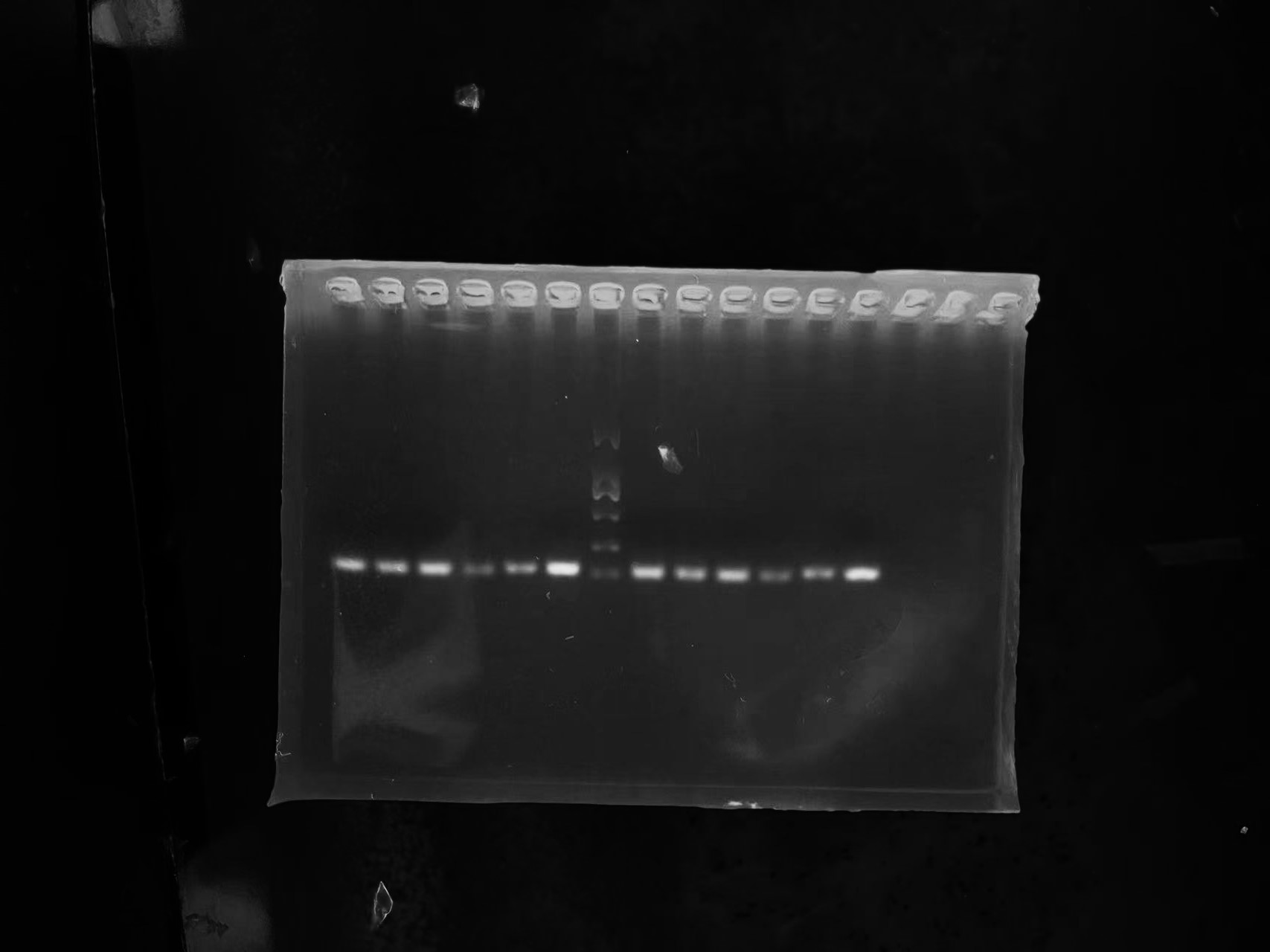

Supplement: Supplementary file 1 — Additional file 1. Supplementary figure 1-3: RNA gel images for subcellular RNA fractionation assay. [file 41021_2024_308_MOESM1_ESM.zip › S Figures/S Figure 3.jpg]
